# La chronologie d’évolution des modes de dépistage et de traitement des néoplasies intra épithéliales du col utérin adoptés au Maroc

**DOI:** 10.11604/pamj.2024.47.13.40063

**Published:** 2024-01-11

**Authors:** Mohamed Zraidi, Mohammed Ibriz

**Affiliations:** 1 Centre de Référence de la Santé Reproductive, Centre Hospitalier Régional, Ispts, Kenitra, Maroc,; 2 Laboratoire de Production Végétales, Animales et Agro-Industrie, Département de Biologie, Faculté des Sciences, Université Ibn Tofaïl, Kenitra, Maroc

**Keywords:** Cancer du col utérin, lésion précancéreuse, dépistage, thermo coagulation, vaccination anti HPV, Cervical cancer, precancerous lesion, screening, thermo coagulation, HPV vaccination

## Abstract

Au Maroc le but du Plan National de Prévention et de Contrôle du Cancer (PNPCC) est de contribuer à la réduction de l´incidence, de la mortalité et de la morbidité imputables au cancer du col de l´utérus (CCU), dont l´objectif général est d´améliorer la prise en charge des femmes atteintes du CCU en instaurant un plan de route organisé de dépistage, de diagnostic précoce et de traitement de ce cancer, et comme objectifs opérationnels une: 1) atteinte d´au moins 30% du taux de couverture annuelle par le dépistage du CCU; 2) atteinte d´au moins 80% du taux de participation au dépistage du CCU par cycle de dépistage; 3) atteinte de 100% du taux de prise en charge des lésions pré malignes du col dépistées dans le cadre du programme. Le dépistage du CCU concerne toutes les femmes âgées de 30 ans à 49 ans révolus. Le test de dépistage actuellement retenu est l´Inspection Visuelle du col utérin à l´Acide acétique (IVA), qui sera suivie si positive d´un examen colposcopique voire une biopsie. L´IVA est effectuée au niveau des centres de soins urbains et ruraux par un personnel de santé qualifié. En sachant que le test utilisé auparavant est le frottis cervico-vaginal. La thermo coagulation, encore appelée cold coagulation est actuellement le traitement de choix des lésions intra épithéliales (LIE) qui sont éligibles à ce traitement, et enfin le programme national a introduit la vaccination anti HPV dans le programme national de vaccination (PNI).

## Abstract (en)

In Morocco, the purpose of the National Cancer Prevention and Control Plan (PNPCC) is to decrease the incidence, mortality, and morbidity attributable to cervical cancer (CC), including the general objective which is to improve women´s care by setting up an organized system for screening, early diagnosis and treatment of this disease, and as operational objectives an: 1) achievement of at least 30% of the annual coverage rate by cervical cancer (CC) screening; 2) achievement of at least 80% of the rate of participation in CC screening per screening cycle; 3) achievement of 100% of the treatment rate for precancerous lesions screened within the framework of the program. CC screening concerns all women aged 30 to 49 years old. Women who have already had CC and pregnant women from the 8^th^ week of amenorrhea until the 6^th^ week postpartum are excluded from the program. The screening test currently used is the naked eye inspection with acetic acid or visual inspection with acetic acid (VIA), which will be followed by a colposcopy exam and biopsy if a precancerous lesion is confirmed. The VIA is carried out at the level of urban and rural health centers, by a trained health professional. Knowing that the pap-smear test was widely used before. Thermo coagulation, also called: cold coagulation, is currently the main treatment for intraepithelial lesions (LIE) that are eligible for this treatment, and finally the national program has introduced anti-HPV vaccination within the national vaccination program (NPI).

## Essay

L´Observatoire Mondial du Cancer (CIRC) estimait, à travers les registres de cancer en population de 185 pays en 2020, à 604 127 cas de CCU et 341831 décès dus à la maladie, plus de 90% surviennent dans les pays à revenus faible ou intermédiaire. Le cancer du col utérin (CCU) se classe parmi les 3 principaux cancers affectant les femmes de moins de 45 ans dans 146 des 185 pays [1-3]. Un fardeau très élevé de la maladie (≥ 15 pour 100000) est enregistré à l'Ouest et au Centre de l´Afrique, en Asie du Sud-Est, en Europe de l'Est, dans les Caraïbes et en Amérique Latine [2-5].

Au Maroc les estimations les plus récentes de l´incidence et de la mortalité par CCU pour l´année 2020 selon le *Global Cancer Observatory* indiquent qu'il existe environ 2165 nouveaux cas (soit un taux d´incidence de 10,4 pour 100000 femmes) [2,6].

**Figure 1:**
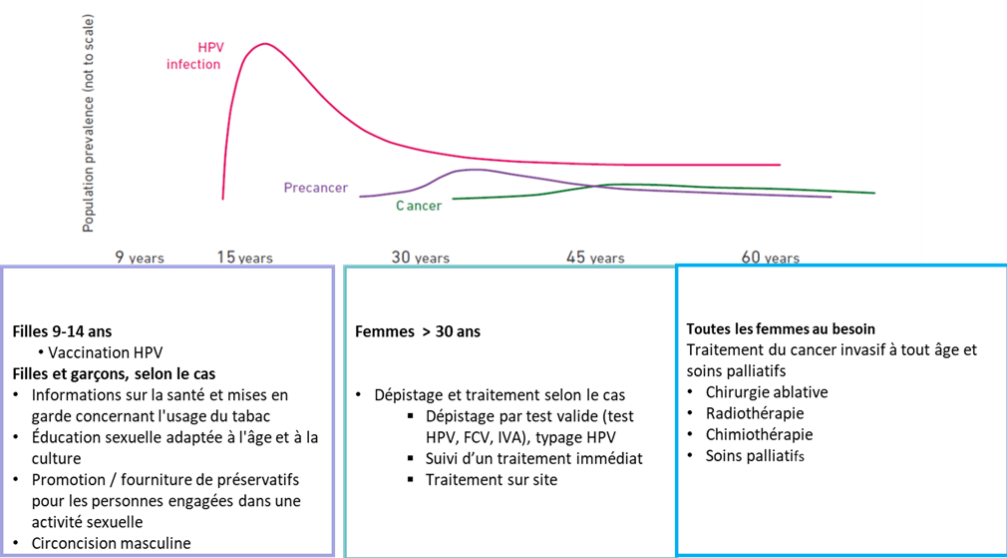
histoire naturelle et approche pour la prévention et le contrôle du cancer du col de l´utérus (CCU)

Le CCU est un cancer rare, car sa lésion précancéreuse persiste pendant de nombreuses années avant de devenir un cancer invasif. Cela permet de détecter et de traiter cette maladie à temps. De toute évidence, l´absence de dépistage et de traitement des néoplasies intra épithéliales cervical (CIN) accroît aussi le risque de progression cancéreuse de l´infection *human papillomavirus infection* (HPV). Si des programmes complets et universels de lutte contre le cancer étaient disponibles pour tous, comprenant notamment une vaccination anti *human papilloma virus (HPV)* pour toutes les jeunes filles et un dépistage et traitement des lésions pré-malignes pour toutes les femmes à risque, cela permettrait de réduire considérablement les cas de CCU [7]. C´est ainsi que notre système de santé nous offre des opportunités pour, premièrement, dépister les lésions précancéreuses du col par l´IVA. Secondairement, les traiter par thermo coagulation, encore appelée: *cold-coagulation*. Et en plus, les prévenir par la vaccination anti HPV récemment introduite dans le Programme national d´immunisation (PNI). En l´occurrence, ces activités sont organisées en trois niveaux de soins (Figure 1) [8].

Le programme de prévention secondaire ou dépistage est basé actuellement sur l´IVA, la colposcopie, et la biopsie. Les activités de détection précoce sont très importantes dans la réduction de l´incidence de certains néoplasies. Dans ce cadre, le PNPCC a retenu parmi ses priorités la détection des néoplasies mammaires et cervicales [9]. En effet, avant 2010, il n'y avait pas de programme de dépistage systématique des cancers au Maroc, en dehors du dépistage individuel réalisé principalement via le FCV. Cela a eu des conséquences néfastes, car environ deux tiers des cas de CCU sont diagnostiqués tardivement. Le frottis cervico-vaginal (*FCV*) était le seul test de dépistage utilisé, certains praticiens continuent d´adopter encore le frottis comme moyen de dépistage malgré sa faible sensibilité par rapport à l´IVA [10,11]; tandis que d´autres prescrivent exceptionnellement le test de génotypage HPV malgré son prix encore élevé. A partir de cette date le MSPS en partenariat avec la Fondation Lalla Salma Prévention et Traitement des Cancers (FLLPTC) a mis en place un programme pilote en partenariat avec la Fondation Lalla Salma Prévention et Traitement des Cancers pour le dépistage du CCU et du sein à la ville de Temara. Après évaluation de ce programme pilote en fin 2011, une généralisation du programme a été lancée en début 2012 dans cinq régions, puis étendue progressivement à l'ensemble du territoire national [12].

Pour consolider les acquis de ce programme, un deuxième plan décennal pour la période 2020-2029 a été mis en place. La détection précoce du CCU a été intégrée dans les activités de santé de la reproduction dans tous les niveaux du système de soins. Le programme s'adresse à toutes les femmes âgées de 30 à 49 ans. Le test de dépistage utilisé est l'IVA, qui est réalisé dans les établissements de soins de santé de base (ESSB) par un professionnel qualifié. Les résultats négatifs doivent être réévalués tous les trois ans. Si une CIN est détectée, elle sera traitée conformément aux recommandations en vigueur.

Concernant le traitement des lésions précancéreuses, avant, les lésions intra-épithéliales (LIE) diagnostiquées sont généralement mises sous: surveillance si cervical intra-épithéliales néoplasia (CIN) I ou traitées par résection à l´anse diathermique (RAD) si CIN II, CIN III. Toutefois, certaines néoplasies intra-épithéliales sont traitées chirurgicalement par une conisation. Actuellement le traitement des lésions précancéreuses de bas grade voir de haut grade (éligible au traitement) du col de l´utérus est basé sur la *cold-coagulation*, introduite récemment dans notre arsenal thérapeutique; et les lésions de haut grade (non éligible au traitement par la cold coagulation) sont traitées par la RAD, par conisation à froid ou autres méthodes thérapeutiques. En effet, selon le PNPCC les indications (critères d´éligibilité) pour l´utilisation de la thermo coagulation sont les suivantes: proposer la thermo coagulation uniquement aux femmes ayant une lésion intraépithéliale du col utérin de bas grade qui correspond à un score de Swede allant de 0 à 5 avec une zone de jonction pavimento-cylindrique de type 1 et dont la superficie est inférieure à 75 % de la surface de l´exocol; est considérée comme non éligible à la thermo coagulation, toute femme ayant, à la colposcopie, une LIE de haut grade correspondant à un score de Swede de 6 et plus et/ou une zone de transformation de type 2 ou 3 et/ou une superficie supérieure à 75% de la surface de l´exocol; réaliser une biopsie obligatoire pour un examen histologique de la lésion à traiter avant toute thermo-coagulation afin de conserver un spécimen; procéder au traitement de la lésion par thermo coagulation directement après la réalisation de la biopsie en respectant les règles de la procédure (technique de pose de l´électrode et temps d´application); délivrer un counseling après la prestation de service; instaurer un suivi chaque six mois la première année, ensuite tous les ans et ce durant toute la vie de la femme ayant reçu un traitement pour une lésion intra-épithéliale du col utérin; les patientes qui vont satisfaire les critères d´éligibilité sus-indiqués vont être traitées par thermo-coagulation par un médecin généraliste ou un médecin gynécologue formés en colposcopie et en *cold coagulation* au niveau du CRSR.

A côté de la cold coagulation récemment adoptée, le tour est venu sur la vaccination anti HPV. Quant au programme de prévention primaire, le vaccin anti HPV a été adopté dans de multiples pays à travers les 5 continents depuis les années 2006/2007 [7-13], la couverture par ce vaccin à ce jour est cependant variable en fonction des zones géographiques et de l´indice de développement humain. En 2020, l´Organisation mondiale de la Santé (OMS) rapporte que moins d'un quart des pays à faible revenu ont introduit le vaccin contre le HPV dans leur calendrier national de vaccination, tandis que plus de 85% des pays à revenu élevé l'ont fait [2-14]. Le pourcentage de pays dotés de programmes réguliers de vaccination anti-HPV a représenté à fin juin 2020 un pourcentage de 31% en Afrique, 40% en Asie, 56% en Océanie, 77% en Europe et 85% aux Amériques [3]. Dans ce sens et en mois de septembre 2022 une circulaire conjointe entre le MSPS et le ministère de l´éducation et de la jeunesse a été lancée annonçant que le MSPS va intégrer le vaccin anti HPV dans le PNI marocain, et ce à partir du mois d´octobre 2022; la vaccination va s´adresser au filles âgées de 11 ans essentiellement celles scolarisées, qui vont recevoir 2 doses séparées au moins de 6 mois [15].

Le PNPCC prévoit une offre de soins en trois niveaux pour la détection précoce et la prise en charge du CCU. Au niveau 1, le dépistage est effectué dans les ESSB, ainsi que dans les centres de référence de la santé reproductive (CRSR) pour la confirmation du diagnostic et la prise en charge thérapeutique des lésions pré malignes du col. Au niveau 2, un bilan d'extension est réalisé et la prise en charge thérapeutique est adaptée en fonction de la gravité des lésions dépistées, avec un suivi des femmes traitées. Enfin, au niveau 3, une prise en charge spécialisée est mise en place en cas de forme de cancer nécessitant un geste chirurgical, une chimiothérapie, une radiothérapie, une hormonothérapie ou des soins palliatifs.

Pour conclure, Le plan national de dépistage et de diagnostic précoce du CCU est institutionnalisé depuis 2012. Le test de dépistage utilise actuellement le test IVA chez les femmes dont l´âge est compris entre 30 à 49 ans. Il est prévu dans le cadre du PNPCC 2020-2029 de passer au dépistage par le test HPV chez les femmes de 30 et 40 ans en application des recommandations de l´OMS qui préconise la nécessité de switcher vers un moyen de dépistage plus efficient, c´est pour cela qu´une étude de la faisabilité technique du dépistage du HPV est récemment lancée dans trois régions marocaines. A cet effet le Maroc a adopté la stratégie préconisée par l´OMS appelée stratégie «90-70-90» [16].

Ainsi est la chronologie de progression de la stratégie d´être aux prises des lésions dysplasiques du col utérin au Maroc.
